# Phasor Histone FLIM-FRET Microscopy Maps Nuclear-Wide Nanoscale Chromatin Architecture With Respect to Genetically Induced DNA Double-Strand Breaks

**DOI:** 10.3389/fgene.2021.770081

**Published:** 2021-12-10

**Authors:** Jieqiong Lou, Ashleigh Solano, Zhen Liang, Elizabeth Hinde

**Affiliations:** ^1^ School of Physics, University of Melbourne, Melbourne, VIC, Australia; ^2^ Department of Biochemistry and Pharmacology, University of Melbourne, Melbourne, VIC, Australia; ^3^ Cancer and RNA Laboratory, St. Vincent’s Institute of Medical Research, Fitzroy, VIC, Australia; ^4^ Department of Medicine, Melbourne Medical School, St Vincent’s Hospital, University of Melbourne, Fitzroy, VIC, Australia

**Keywords:** DNA repair, chromatin, histones, fluorescence lifetime imaging microscopy (FLIM), Förster resonance energy transfer (FRET)

## Abstract

A DNA double-strand break (DSB) takes place in the context of chromatin, and there is increasing evidence for chromatin structure to play a functional role in DSB signaling and repair. Thus, there is an emerging need for quantitative microscopy methods that can directly measure chromatin network architecture and detect changes in this structural framework upon DSB induction within an intact nucleus. To address this demand, here we present the phasor approach to fluorescence lifetime imaging microscopy (FLIM) of Förster resonance energy transfer (FRET) between fluorescently labeled histones in the DSB inducible via AsiSI cell system (DIvA), which has sufficient spatial resolution to map nuclear-wide chromatin compaction at the level of nucleosome proximity with respect to multiple site-specific DSBs. We also demonstrate that when phasor histone FLIM-FRET is coupled with immunofluorescence, this technology has the unique advantage of enabling exploration of any heterogeneity that exists in chromatin structure at the spatially distinct and genetically induced DSBs.

## Introduction

Inside the nucleus of a living cell, DNA is folded around histone proteins into nucleosomes and compacted into a multi-layered three-dimensional (3D) structure called chromatin (Luger et al., 2012; Bickmore, 2013; Bonev and Cavalli, 2016). At any moment in time, a DNA double-strand break (DSB) can occur anywhere within this dynamic structural framework, and somehow, a cellular surveillance system termed the “DNA damage response” (DDR) (Jackson and Bartek, 2009) has the capacity to instantaneously detect DSB induction and recruit repair machinery to this type of genetic damage (Kalousi and Soutoglou, 2016; Hauer and Gasser, 2017; Marnef and Legube, 2017). Initially, chromatin was viewed as an obstacle to DSB repair that the DDR must first “open” and then restore upon DSB resolution. More recently, however, it has become apparent that the chromatin compaction status of a DSB plays a more active role in DNA damage signaling and DSB repair pathway choice (Soria et al., 2012; Lemaître et al., 2014; Clouaire and Legube, 2015; Polo and Almouzni, 2015). Local reorganization in chromatin network architecture has been shown to spatiotemporally modulate the arrival and retention of different DNA repair factors at DSB sites (Hinde et al., 2014; Smith et al., 2019). Thus, in order to understand how genome integrity is maintained at a cellular level, there is an emerging need to study DSB repair within the context of chromatin and the 3D nuclear landscape of a living cell.

The chromatin “opening” and “compacting” events that follow DSB induction (Klement et al., 2014; Kalousi et al., 2015; Thorslund et al., 2015; Luijsterburg et al., 2016) are underpinned by nanoscale changes in the spacing between nucleosomes (Hauer and Gasser, 2017), and these dynamics occur on a spatial scale that is well below the diffraction limit of optical microscopy (Luger and Hansen, 2005; Ou et al., 2017; Ohno et al., 2018; Ochs et al., 2019; Whelan and Rothenberg, 2021). Thus, with the aim of rendering any DDR-induced changes to a local chromatin structure visible in a living cell, we recently demonstrated that Förster resonance energy transfer (FRET) between fluorescently labeled histones is a sensitive real-time readout of nucleosome proximity during DSB repair (Lou et al., 2019) that can be spatially mapped throughout the nucleoplasm by the phasor approach to fluorescence lifetime imaging microscopy (FLIM) (Liang et al., 2020). From coupling FLIM detection of FRET between histone H2B tagged to eGFP (H2B-eGFP) and mCherry (H2B-mCh) with DSB induction via near-infrared (NIR) laser micro-irradiation, we quantified a rapid chromatin decompaction event central to a DNA repair locus that was surrounded by a border of compact chromatin foci and found this chromatin structure to be critical for the timely accumulation of DNA repair factors at a DSB site (Lou et al., 2019). Thus, this phasor histone FLIM-FRET assay has the potential to be an invaluable tool for biologists studying DSB repair, since it has sufficient spatiotemporal resolution to reveal what is normally an invisible layer of regulation to a cellular DDR.

Here in this study, we demonstrate the capacity of the phasor histone FLIM-FRET assay to spatially map chromatin architecture with respect to DNA damage in the DSB inducible via AsiSI cell system (DIvA) (Iacovoni et al., 2010). DIvA cells harbor a 4-hydroxytamoxifen (4OHT)-inducible AsiSI restriction enzyme that allows for induction of approximately 100 site-specific DSBs throughout the genome upon 4OHT treatment (Iacovoni et al., 2010; Massip et al., 2010; Aymard et al., 2014). Thus, by multiplexing phasor histone FLIM-FRET with immunofluorescence (IF) against phosphorylated serine 139 of histone variant 2AX (γH2AX), we are able to spatially map nuclear-wide chromatin compaction at the level of nucleosome proximity with respect to DIvA DSB locations. From image analysis of this three-color experiment across multiple DIvA nuclei, we find in agreement with our previous study employing NIR laser micro-irradiation (Lou et al., 2019) that DSB induction induces a global chromatin compaction event that surrounds sites of DNA damage, which statistically represent nuclear locations that are in a more “open” chromatin state. While a benefit of NIR laser micro-irradiation as a method for DSB induction was temporal resolution, an important advantage of the DIvA cell system is having access to the spatial heterogeneity that underlies this quantified chromatin response. Thus in a final experiment, to demonstrate this utility, we perform a four-color experiment that enables the chromatin structure reported by histone FRET to be studied as a function of the DSB repair pathway. We anticipate that this unique capacity of the phasor histone FLIM-FRET assay in DIvA alongside IF has the potential to facilitate discovery into how exactly chromatin structure regulates a DSB DNA damage response.

## Results

**FIGURE 1:**
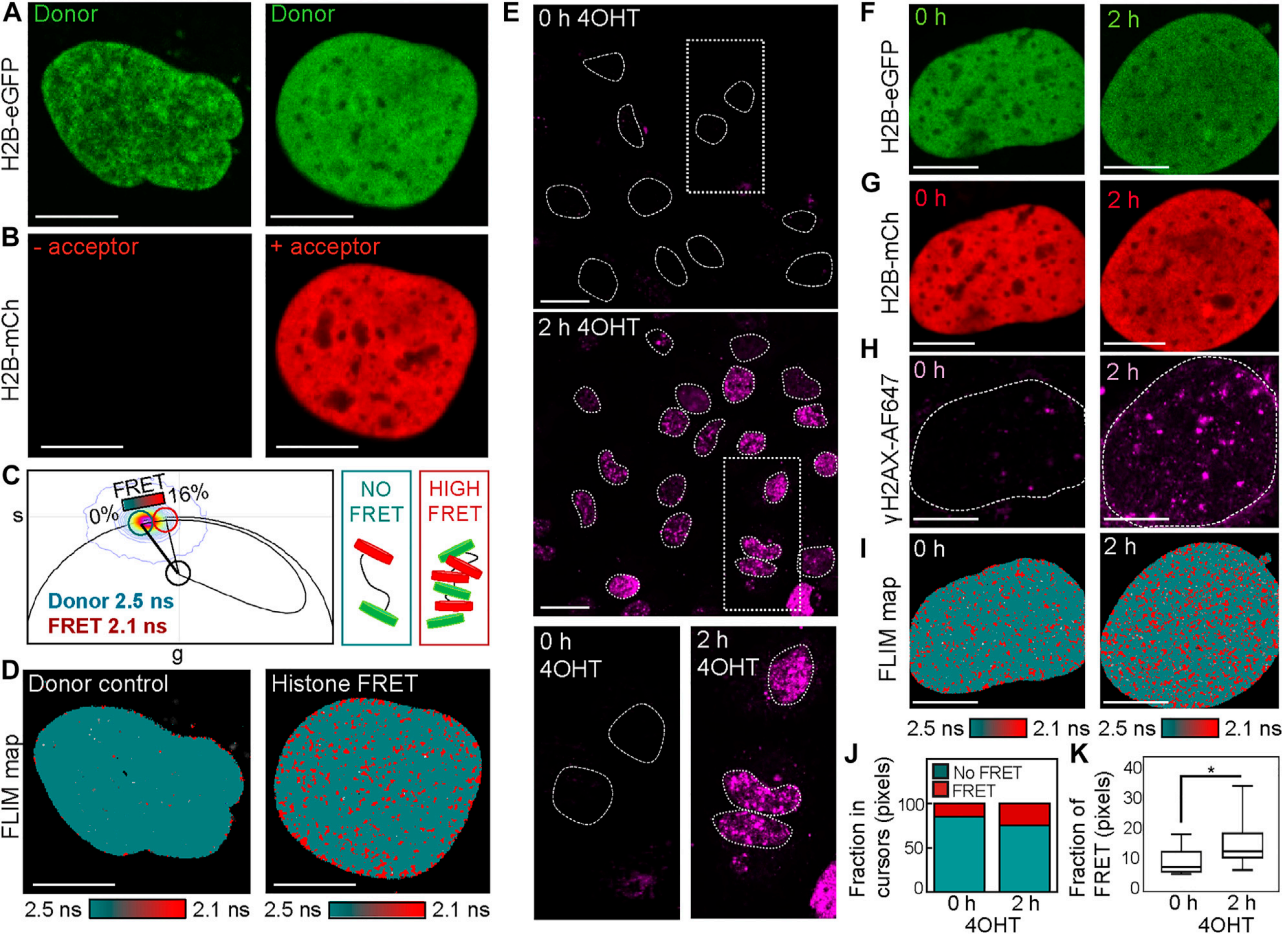
Phasor histone FLIM-FRET coupled with γH2AX IF reveals DSB induction in the DIvA cell system to induce nuclear-wide chromatin compaction. **(A,B)** Fixed DIvA nuclei expressing H2B-eGFP **(A)** in the absence **(left)** versus presence **(right)** of H2B-mCh **(B)** (scale bar 10 μm). **(C)** Phasor distribution of H2B-eGFP in the absence (donor control) versus presence of H2B-mCh (histone FRET experiment) with a theoretical FRET trajectory superimposed (black curve) that extends from the unquenched donor lifetime (teal cursor, 2.5 ns). This FRET trajectory enables characterization of the histone FRET efficiency as 16% (red cursor, 2.1 ns) and definition of a palette to detect open (teal) versus compact (red) chromatin. **(D)** FLIM maps of H2B-eGFP in the absence **(left)** versus presence **(right)** of H2B-mCh pseudo-colored according to the FRET palette defined in the phasor plot of panel **(C)**. **(E)** IF against γH2AX in DIvA before versus after 2 h of treatment with 4OHT (scale bar 20 μm). **(F–H)** Fixed DIvA nuclei co-expressing the histone FRET pair H2B-eGFP **(F)** and H2B-mCh **(G)** with IF against γH2AX **(H)** in the absence **(left)** versus presence **(right)** of 2 h of treatment with 4OHT (scale bar 10 μm). **(I)** FLIM maps of the cells presented in panels **(F–H)** pseudo-colored according to the FRET palette defined in panel **(C)**. **(J)** Quantification of the fraction of pixels in the phasor cursor that reports no FRET (open chromatin) versus histone FRET (compact chromatin) in the cells presented in panel **(I)**. **(K)** Quantification of the fraction of pixels in the phasor cursor that reports histone FRET (compact chromatin) across multiple cells before versus after 2 h of 4OHT treatment (N = 11 and 47 cells, respectively, three biological replicates). The box and whisker plot shows the minimum, maximum, and sample median. *p < 0.05 (unpaired *t*-test).

Phasor histone FLIM-FRET microscopy coupled with IF maps nuclear-wide changes in chromatin compaction with respect to DSB induction in the DIvA cell system. To quantify the local versus global chromatin compaction status of nucleus architecture with respect to multiple site-specific DSBs, here we combine phasor histone FLIM-FRET analysis with IF of γH2AX in the DIvA cell system. FRET is an optical phenomenon that reports fluorescent protein–protein interaction on a scale of 1–10 nm, and in the context of chromatin labeled with donor–acceptor fluorescent histones (Llères et al., 2009), FRET reports nucleosome proximity with nanoscale resolution. Thus, to implement histone FRET in the DIvA cell system, we first transfected DIvA cells with H2B tagged to eGFP (H2B-eGFP) in the absence (donor control) versus presence of mCherry (H2B-mCh) (Figures 1A,B). Then in fixed and washed DIvA nuclei expressing the donor control versus donor–acceptor FRET pair, we acquired FLIM data in the H2B-eGFP (donor) channel where quenching of the donor lifetime in the presence of H2B-mCh (acceptor) reports histone FRET (Figures 1C,D). Quantification of this donor control versus histone FRET experiment in the DIvA cell system by the phasor approach to lifetime analysis enabled the FRET efficiency of compact chromatin to be characterized as 16% (i.e., donor lifetime shift from 2.5 to 2.1 ns) (Figure 1C) and definition of a cursor-based palette to spatially map compact (red pixels) versus open chromatin (teal pixels) throughout DIvA nuclei (Figure 1D).

To next employ histone FRET as a readout of chromatin network architecture with respect to sites of DSB induction in the DIvA cell system, we first confirmed via IF for γH2AX Alexa Fluorophore 647 (γH2AX-AF647) in DIvA cells fixed 2 h after 4-hydroxytamoxifen (4OHT) treatment that multiple DSBs do form across the genome (Figure 1E). Then from careful design of a multi-colored imaging experiment that aimed to measure histone FRET between H2B-eGFP and H2B-mCh (Figures 1F,G) in the presence of γH2AX-AF647 IF (Figure 1H), we spatially mapped compact versus open chromatin in the presence versus absence of multiple DSB foci (Figure 1I) without artifact from 4OHT addition (Supplementary Figure S1). Quantification of this multiplexed imaging experiment via calculation of the fraction of pixels exhibiting histone FRET (our readout of a compact chromatin state) (Figure 1J) revealed genetic DSB induction to initiate significant nuclear-wide chromatin compaction when applied across multiple cells (Figure 1K). This result alongside a qualitative comparison of γH2AX-AF647 localization with histone FRET after 4OHT treatment (Figures 1H,I, right) suggested DSB sites to occupy the few “open” chromatin regions that exist within the detected nuclear-wide chromatin compaction event. Thus, to further investigate this observation, we next performed a γH2AX-AF647–based mask analysis of the histone FRET maps derived after 4OHT treatment, to enable quantification of the local (inside the DSB site) versus global (outside the DSB site) chromatin response to DSB induction.

**FIGURE 2:**
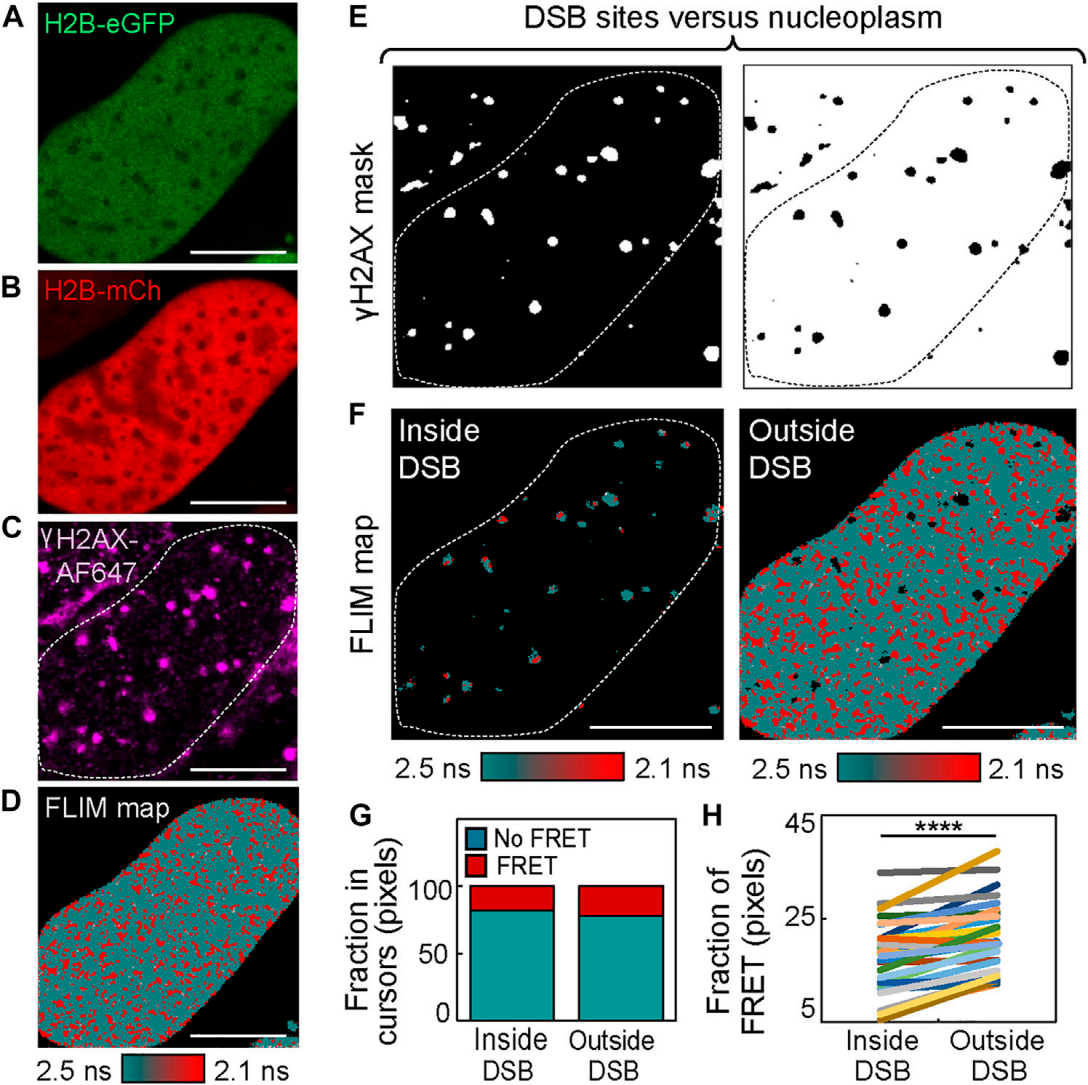
IF-based mask analysis of histone FRET in the DIvA cell system reveals chromatin to be “open” at sites of DSB induction. **(A–C)** DIvA nucleus co-expressing the histone FRET pair H2B-eGFP **(A)** and H2B-mCh **(B)** that has been fixed with IF against γH2AX **(C)** after 2 h of treatment with 4OHT (scale bar 10 μm). **(D)** FLIM map of the cell presented in panels **(A–C)** pseudo-colored to report histone FRET (red pixels) versus non-FRET (teal pixels). **(E)** Masks based on γH2AX IF presented in panel **(C)** that select chromatin inside **(left)** versus outside **(right)** of DIvA DSBs. **(F)** Pseudo-colored histone FRET maps with threshold defined by masks presented in panel **(E)** applied to select inside **(left)** versus outside **(right)** of DSBs. **(G)** Fraction of pixels reporting FRET (compact chromatin) versus no FRET (open chromatin) within masked FLIM maps presented in panel **(F)**. **(H)** Quantitation of the fraction of histone FRET (compact chromatin) inside versus outside of DSBs after 2 h of 4OHT treatment across multiple cells (N = 29 cells, three biological replicates). The box and whisker plot shows the minimum, maximum, and sample median. ****p < 0.0001 (paired *t*-test).

To generate a mask that enables histone FRET analysis of chromatin compaction inside versus outside of DSB foci (Figures 2A–D), a threshold based on γH2AX-AF647 IF was employed (Figure 2E). This binary mask allowed for selection of pixels within the FLIM map that occupy DSB sites versus the surrounding nucleoplasm (Figure 2F), and quantitation of the fraction of pixels exhibiting histone FRET in either environment (Figure 2G). From application of this analysis to multiple cells after 4OHT treatment (Figure 2H), we confirmed DSB sites to statistically be in a more “open” chromatin state than the surrounding chromatin environment, which was compacted upon DSB induction (Figure 1). Interestingly, this differentially regulated reorganization in local versus global chromatin structure that was induced by multiple DSBs being genetically cut at distinct nuclear locations is in direct agreement with our previous study, which coupled histone FRET with NIR laser micro-irradiation to cut multiple DSBs at a single nuclear location (Lou et al., 2019). Thus, while NIR laser micro-irradiation was advantageous in terms of temporal resolution and enabling observation of early changes in DSB chromatin structure, a clear advantage of the DIvA cell system for histone FRET assessment of DSB chromatin during repair is the potential for this assay to explore any spatial heterogeneity that underlies this response.

**FIGURE 3:**
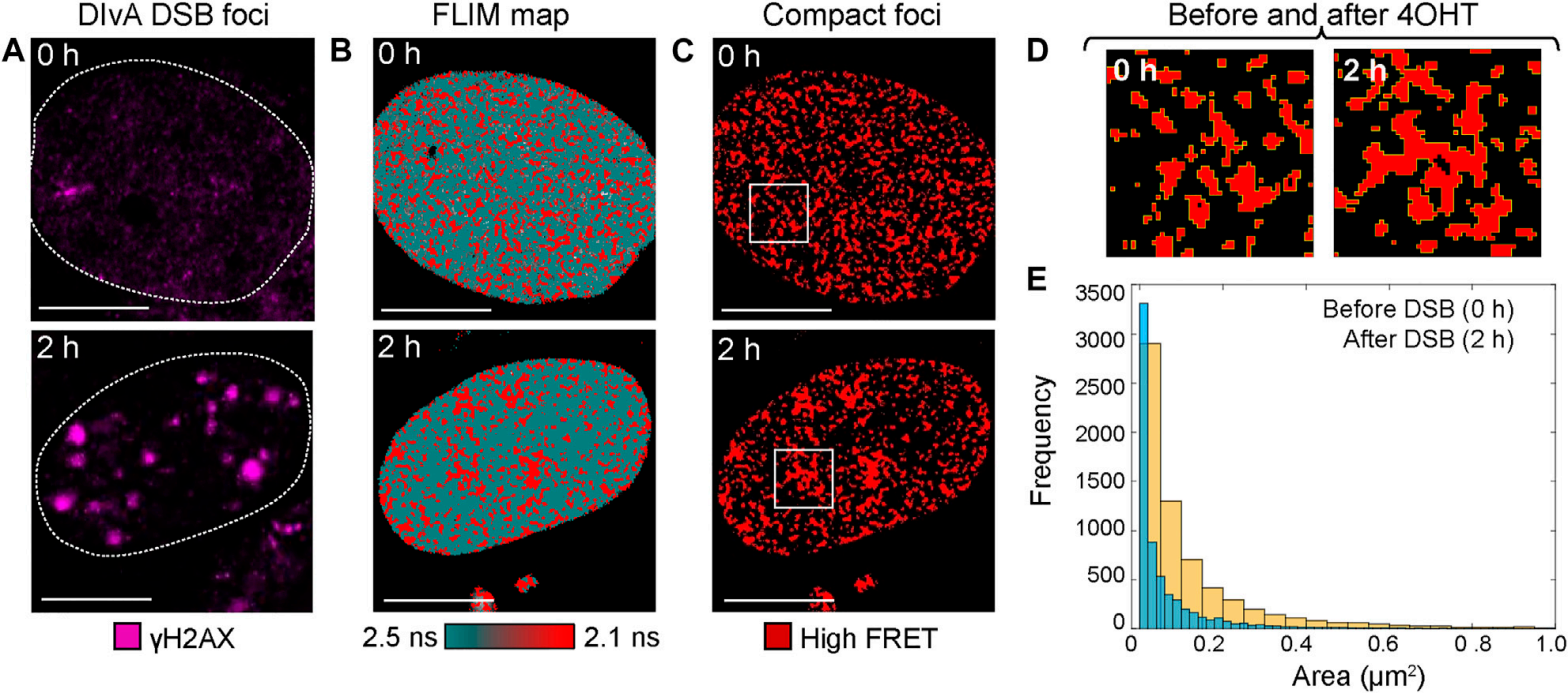
Particle analysis of histone FRET in DIvA cell FLIM maps reveals sub-micron changes in compact chromatin network organization upon DSB induction. **(A)** DIvA nucleus expressing the histone FRET pair (H2B-eGFP and H2B-mCh) that has been fixed with IF against γH2AX before **(top)** versus after **(bottom)** 2 h of treatment with 4OHT (scale bar 10 μm). **(B)** FLIM maps of the cells presented in panel **(A)** pseudo-colored to report histone FRET (red pixels) versus no FRET (teal pixels). **(C)** Localization of compact chromatin foci extracted from the histone FRET maps presented in panel **(B)**. **(D)** Zoom of region of interest (white box) in localization maps presented in panel **(C)**. **(E)** Histogram of the size of compact chromatin foci as detected by histone FRET in DIvA before (blue) versus after 2 h (yellow) of treatment with 4OHT (N = 6 cells, two biological replicates).

IF-guided image analysis of phasor histone FLIM-FRET microscopy data acquired in DIvA cells quantifies chromatin network organization and enables exploration of DSB foci heterogeneity. To demonstrate the potential of phasor histone FLIM-FRET microscopy and IF in DIvA cells to enable both 1) a quantitative insight into the nuclear-wide spatial organization of compact chromatin with respect to DSBs and 2) exploration of heterogeneity in the local chromatin response at DSBs, here we performed two types of image analysis to acquired FLIM maps of histone FRET. The first type of analysis extracts the nuclear-wide localization of high FRET compact chromatin foci within a FLIM map, treats them as particles, and then quantifies their spatial distribution in terms of particle size. From application of this analysis to DIvA nuclei that were untreated versus treated with 4OHT (Figures 3A,B), we find the extracted network of high FRET compact chromatin foci (Figure 3C), to undergo a spatial reorganization in response to DSB induction that results in an increase in foci area (Figures 3D,E). This result, alongside the finding that DSB induction initiates a nuclear-wide chromatin compaction event at the level of nucleosome proximity (Figures 1, 2), suggests that, in addition to this global but nanoscale reorganization in chromatin structure, which occurs outside of DSB sites, a DSB DNA damage response also initiates sub-micron changes to higher order chromatin network organization (Figure 3).

**FIGURE 4:**
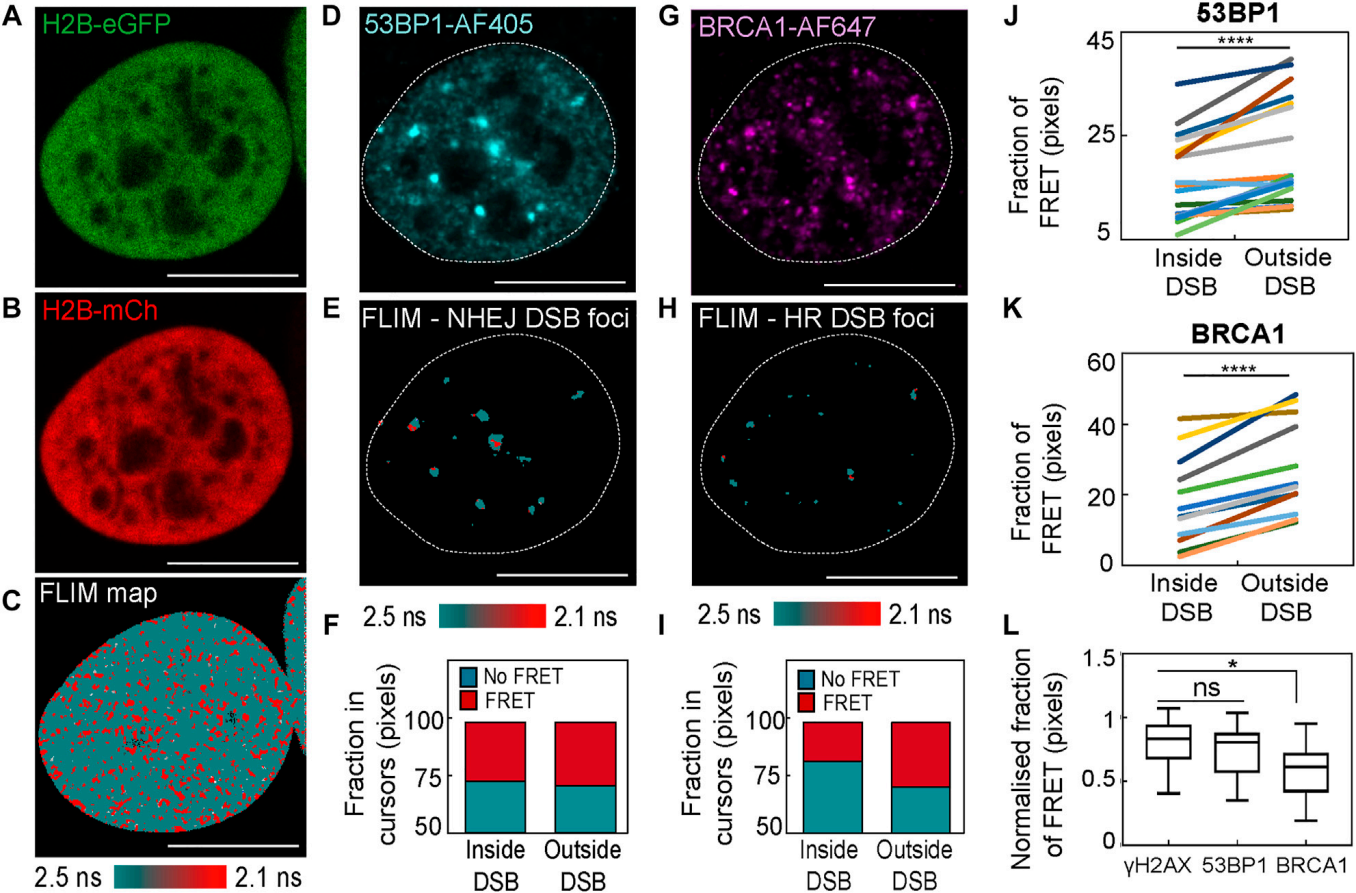
An IF-based mask analysis of DSB histone FRET heterogeneity in the DIvA cell system. **(A,B)** DIvA nucleus co-expressing the histone FRET pair H2B-eGFP **(A)** and H2B-mCh **(B)** that has been treated with 4OHT for 2 h and fixed with IF against different DNA repair proteins. **(C)** FLIM map of the cell presented in panels **(A,B)** pseudo-colored to report histone FRET (red pixels). **(D)** IF against NHEJ DNA repair protein 53BP1 (53BP1-AF405) in the cell presented in panels **(A–C)**. **(E,F)** Pseudo-colored histone FRET map from panel **(C)** with mask based on the 53BP1-AF405 signal **(D)** applied to select NHEJ DSB foci **(E)** and the fraction of pixels reporting histone FRET (red pixels) within versus outside of this mask **(F)**. **(G)** IF against HR DNA repair protein BRCA1 (BRCA1-AF647) in the cell presented in panels **(A–C)**. **(H,I)** Pseudo-colored histone FRET map from panel **(C)** with mask based on the BRCA1-AF647 signal **(G)** applied to select HR DSB foci **(H)** and the fraction of pixels reporting histone FRET (red pixels) within versus outside of this mask **(I)**. **(J)** Quantification of the fraction of histone FRET (compact chromatin) inside versus outside of 53BP1 identified NHEJ DSBs after 2 h of 4OHT treatment across multiple nuclei (N = 18 cells, two biological replicates). **(K)** Quantification of the fraction of histone FRET (compact chromatin) inside versus outside of BRCA1 identified HR DSBs after 2 h of 4OHT treatment across multiple nuclei (N = 12 cells, two biological replicates). **(L)** A quantitative comparison of the fraction of histone FRET (compact chromatin) inside γH2AX labeled foci (all DSBs) versus 53BP1 foci (NHEJ DSBs) and BRCA1 foci (HR DSBs) normalized to the fraction of histone FRET in the surrounding nucleoplasm (N ≥ 12 cells, two or three biological replicates). The box and whisker plot shows the minimum, maximum, and sample median. In **(J,K)**, ****p < 0.0001 (paired *t*-test). In **(L)**, *p < 0.05 and ns > 0.05 (unpaired *t*-test).

To next investigate heterogeneity in the local chromatin response reported by histone FRET at DIvA DSB sites, we performed IF against not only γH2AX, which is expected to highlight the total population of DSBs present, but also different DNA repair proteins that highlight the DSB sub-population set to undergo repair by one of two dominant DSB repair pathways. In particular, we performed an IF-guided mask analysis of histone FRET maps acquired in DIvA nuclei co-expressing H2B-eGFP and H2B-mCherry, which were treated with 4OHT for 2 h (Figures 4A–C) and fixed with IF against 1) tumor suppressor p53 binding protein 1 (53BP1-AF405) that highlights DSBs marked for non-homologous end joining (NHEJ) (Figures 4D–F) and 2) breast cancer type 1 susceptibility protein (BRCA1-AF647) that highlights DSBs marked for homologous recombination (HR) (Figures 4G–I). Collectively, these experiments enabled quantification of chromatin compaction inside versus outside of DSB foci marked for NHEJ (Figure 4J) and HR (Figure 4K), as well as investigation into whether NHEJ versus HR DSB repair takes place in different chromatin environments (Figure 4L).

We find from this analysis that both NHEJ and HR DSB sites are statistically in a more “open” chromatin state than their surrounding undamaged chromatin environment (Figures 4J, K), which is in keeping with our γH2AX-guided analysis (Figure 2H). Also, intriguingly, if we take into account the baseline chromatin compaction status of each DIvA nucleus analyzed (i.e., normalized with respect to FRET fraction in pixels outside DSB sites), we find that while 53BP1 DSB foci marked for NHEJ are not significantly different from γH2AX DSB foci, BRCA1 DSB foci marked for HR are statistically more “open” than γH2AX DSB foci (Figure 4L). The molecular mechanism and physiological function of why HR DSB foci are more “open” needs to be further investigated; however, it is in keeping with previous studies that link BRCA1 with roles in chromatin de-condensation (Bochar et al., 2000; Ye et al., 2001), and it does suggest that heterogeneity in terms of chromatin structure does exist as a function of the DSB repair pathway.

## Discussion

In this study, we applied phasor histone FLIM-FRET microscopy to the measurement of nuclear-wide chromatin compaction at the level of nucleosome proximity and demonstrated that this assay can quantify sub-micron changes in the spatial organization of this nanoscale feature upon DSB induction in the DIvA cell system. From coupling this technology with immunofluorescence against histone modifications that highlight DSB sites (e.g., γH2AX) and DNA repair proteins involved in DSB resolution (e.g., 53BP1 and BRCA1), we also highlight the capacity of phasor histone FLIM-FRET to explore spatial heterogeneity in the local DSB chromatin structure as a function of DSB repair pathway choice—NHEJ versus HR. In doing so, we found that DIvA DSBs induce a global chromatin network compaction event that reduces the average spacing between nucleosomes and reorganizes them into larger clusters, in parallel with the local opening of chromatin at DSB sites—especially those marked for repair via HR. Interestingly, this result, which stems from multiple site-specific DSBs being induced at distinct locations throughout the DIvA nucleoplasm, is in strong agreement with our previous study that implemented phasor histone FLIM-FRET in HeLa cells exposed to NIR laser micro-irradiation, which induces multiple DSBs at a single nuclear location (Lou et al., 2019). Thus, chromatin “opening” at a DSB site alongside chromatin compacting of the surrounding DNA appears to be a universal mechanism for efficient repair of DSBs whether they be induced genetically or by a source of radiation.

The next question is the following: What biological function do these detected changes in chromatin structure serve for DSB resolution? In the context of DNA repair, there is already evidence obtained via super-resolution microscopy that a nanoscale reorganization in the chromatin structure regulates DNA repair protein access and retention at DSB sites (Ochs et al., 2019; Whelan and Rothenberg, 2021). Along this line, in our previous study that employed NIR laser micro-irradiation, we found that the compacted chromatin boundary of a DSB repair locus serves to modulate the mobility and access of the NHEJ repair factor tumor suppressor 53BP1 to the central “opened” region of this type of genomic lesion (Lou et al., 2019). Thus, given the demonstrated potential of the histone FRET assay to explore DSB chromatin structure, here as a function of DNA repair pathway choice when coupled with IF in DIvA, future experiments will be dedicated toward bettering understanding what is the role of DSB chromatin structure in controlling 53BP1 versus BRCA1 access and identifying whether chromatin plays a role in the decision to proceed toward DSB resolution via NHEJ versus HR.

## Materials and Methods

### Cell Culture, Transient Transfection, and IF

DIvA cells (originally provided by Gaëlle Legube, LBCMCP, CNRS, Toulouse, France) were grown in Dulbecco’s modified Eagle’s medium (Lonza) supplemented with 10% bovine growth serum (Gibco), 1x Pen-Strep (Lonza), and 1 μg/ml puromycin (Thermo Fisher Scientific) at 37°C in 5% CO_2_. DIvA cells were then plated 24 h before transfection onto 35 mm glass bottom dishes and transiently transfected with H2B-eGFP and H2B-mCherry via use of Lipofectamine 3000 according to the manufacturer’s protocol. Transiently transfected DIvA cells were then treated (or left untreated) with 300 nM of 4OHT for 2 h and then fixed with 4% paraformaldehyde for 15 min, permeabilized with 1 mg/ml Triton X-100 for 15 min at room temperature, and blocked with 1% bovine serum albumin for 30 min. Three rounds of washing with phosphate-buffered saline (PBS) were performed in between each of these fixation steps. For IF against γH2AX (S139) (Catalog number 9718S, Cell Signaling), 53BP1 (Catalog number 4937S, Cell Signaling), and BRCA1 (Catalog number SAB2702136-100UL, Sigma), the fixed DIvA cells were incubated with primary antibody (1:200) overnight at 4°C and then secondary antibody labeled with Alexa Fluor 405 (AF405) or Alexa Fluor 647 (AF647) for 1 h at room temperature. The three rounds of washing step with PBS were also performed in between each of these IF steps. In general, PBS washing not only was critical for fixation and IF but also counteracted a 4OHT-induced shift in the fluorescence lifetime of H2B-eGFP that was unrelated to histone FRET (Supplementary Figures S1A–E).

### Confocal Laser Scanning Microscopy and FLIM Data Acquisition

All fixed cell microscopy measurements were performed on an Olympus FV3000 laser scanning microscope coupled to a 488 nm pulsed laser operated at 80 MHz and an ISS A320 FastFLIM box. A ×60 water immersion objective 1.2 NA was used for all experiments, and the cells were imaged at room temperature. Prior to acquisition of FLIM data in the donor channel (H2B-eGFP) for histone FRET analysis, multi-channel intensity images (two-, three-, and four-color) were acquired from each selected DIvA nucleus to verify that the FRET acceptor (H2B-mCh) was present in excess of H2B-eGFP (i.e., acceptor–donor ratio > 1) and to record the localization of DSB breaks labeled with either H2AX (γH2AX-AF647) or 53BP1 (53BP1-AF405) and BRCA1 (BRCA1-AF647). This involved sequential imaging of a two-phase light path in the Olympus FluoView software. The first phase was set up to image H2B-eGFP and H2B-mCh via use of solid-state laser diodes operating at 488 and 561 nm, respectively, with the resulting signal being directed through a 405/488/561/6033 dichroic mirror to two internal GaAsP photomultiplier detectors set to collect 500–540 nm and 600–700 nm. The second phase was set up to image 53BP1-AF405 and BRCA1-AF647 or just γH2AX-AF647 via use of solid-state laser diodes operating at 405 and 633 nm, respectively, with the resulting signal being directed through a 405/488/561/633 dichroic mirror to two internal GaAsP photomultiplier detectors set to collect 420–460 nm and 600–700 nm. Then in each DIvA nucleus selected, a FLIM map of H2B-eGFP was imaged within the same field of view (256 × 256-pixel frame size, 20 µs/pixel, 90 nm/pixel, 20 frame integration) using the ISS VistaVision software. This involved excitation of H2B-eGFP with an external pulsed 488 nm laser (80 MHz) and the resulting signal being directed through a 405/488/561/633 dichroic mirror to an external photomultiplier detector (H7422P-40 of Hamamatsu) that was fitted with a 520/50 nm bandwidth filter. The donor signal in each pixel was then subsequently processed by the ISS A320 FastFLIM box data acquisition card to report the fluorescence lifetime of H2B-eGFP. All FLIM data were pre-calibrated against fluorescein at pH 9 which has a single exponential lifetime of 4.04 ns.

### FLIM-FRET Analysis

The fluorescence decay recorded in each pixel of an acquired FLIM image was quantified by the phasor approach to lifetime analysis (Digman et al., 2008; Hinde et al., 2012). As described in previously published papers (Hinde et al., 2012; Liang et al., 2020), this results in each pixel of a FLIM image giving rise to a single point (phasor) in the phasor plot, which when used in the reciprocal mode enables each point in the phasor plot to be mapped to each pixel of the FLIM image. Since phasors follow simple vector algebra, it is possible to determine the fractional contribution of two or more independent molecular species coexisting in the same pixel. For example, in the case of two independent species, all possible weightings give a phasor distribution along a linear trajectory that joins the phasors of the individual species in pure form. While in the case of a FRET experiment, where the lifetime of the donor molecule is changed upon interaction with an acceptor molecule, the realization of all possible phasors quenched with different efficiencies describes a curved FRET trajectory in the phasor plot that follows the classical definition of FRET efficiency.

In the context of the histone FRET experiments presented, the phasor coordinates (g and s) of the unquenched donor (H2B-eGFP) and background (cellular autofluorescence) were first determined independently in fixed DIvA cells transfected versus un-transfected with H2B-eGFP. This enabled definition of a baseline from which a FRET trajectory could be extrapolated and then used to determine the dynamic range of FRET efficiencies that describe chromatin network organization in the DIvA cell system (Lou et al., 2019; Liang et al., 2020). From superimposition of this FRET trajectory with the combined phasor distribution measured for H2B-eGFP in fixed DIvA cells co-transfected with H2B-mCh, we find the DIvA chromatin network to exhibit compaction states that range from 0 to 16% in FRET efficiency. This corresponds to a shift in the H2B-eGFP donor lifetime from approximately 2.5 ns (g = 0.39  ±  0.05, s = 0.49 ±  0.05) to 2.1 ns (g = 0.47  ±  0.05, s = 0.50  ±  0.05). We therefore defined two cursors centered at these phasor coordinates to spatially map where chromatin is open (teal cursor) versus compact (red cursor) throughout a FLIM data acquisition in a fixed DIvA nucleus. Also, to quantify the extent to which DIvA chromatin was compacted before versus after DSB induction across multiple nuclei, we calculated the fraction of pixels counted as compact (i.e., FRET state in red cursor). All FLIM-FRET quantification was performed in the SimFCS software developed at the LFD.

### DSB Foci Segmentation and Foci FLIM-FRET Analysis

To quantify the local chromatin structure of DSB foci versus the undamaged nuclear-wide chromatin architecture in DIvA nuclei, we applied an intensity threshold mask based on a DSB protein IF intensity image to FLIM maps pseudo-colored according to histone FRET (compact chromatin) versus no FRET (open chromatin). This involved 1) smoothing each DIvA nucleus’ IF image of DSB localization (i.e., γH2AX-AF647, 53BP1-AF405, or BRCA1-AF647) with a 3 × 3 spatial median filter, 2) transforming this smoothed image into a binary mask based on an intensity threshold that was sufficiently harsh to reject non-specific IF staining but retain DSB foci, 3) applying the IF-guided mask to its associated FLIM map pseudo-colored according to histone FRET, and 4) quantification of the fraction of compact chromatin within (i.e., DSB foci) versus outside (i.e., nucleoplasm) the IF-guided mask.

### Compact Chromatin Foci Size Analysis

To quantify the size of compact chromatin foci detected within a FLIM map pseudo-colored according to histone FRET (compact chromatin) versus no FRET (open chromatin), a binary image of compact chromatin foci was exported from the software SimFCS to ImageJ, and then a particle analysis routine was applied that identified particles based on the following criteria: 1) particle size was from 0 to infinity, 2) all adjacent non-zero pixels were considered one particle, and 3) holes inside connected pixels were considered part of the identified particle. The area of identified particles was calculated as the number of pixels times the area of a single pixel.

### Statistics and Figure Preparation

Statistical analysis was performed by using GraphPad Prism software. Figures were prepared by using Adobe Illustrator, Microsoft PowerPoint, SimFCS, and ImageJ.

## Data Availability

The raw data supporting the conclusions of this article will be made available by the authors, without undue reservation.
