# Supplementary material for: Phasor Histone FLIM-FRET Microscopy Maps Nuclear-Wide Nanoscale Chromatin Architecture With Respect to Genetically Induced DNA Double-Strand Breaks
Source: Front Genet. 2021 Dec 10;12:770081. doi: 10.3389/fgene.2021.770081 (PMC8702996; doi:10.3389/fgene.2021.770081)
Supplement: Supplementary file 1 [file DataSheet1.PDF]

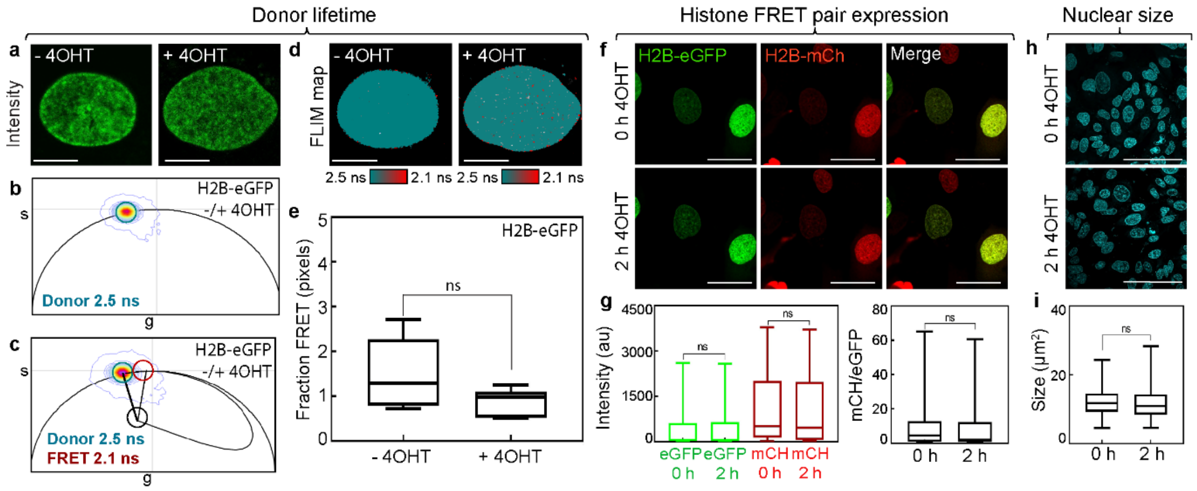

Supplementary Figure 1. DSB induction in the DIvA cell line via use of 4OHT does not impact phasor FLIM analysis, histone FRET expression or nuclear size. a. Intensity images of DIvA nuclei expressing H2B-eGFP in the absence (left) versus presence (right) of 2 h of 4OHT treatment after fixation and washing with phosphate buffered saline (PBS) (Scale bar 10 µm). **b-c.** Phasor distribution of H2B-eGFP in the absence versus presence of 2 h of 4OHT treatment after fixation and washing with PBS is centred at 2.5 ns (teal cursor) (b) and not right shifted along the FRET trajectory (c) into the FRET state (red cursor) that is observed when H2B-mCH is present. **d**. FLIM maps of the H2B-eGFP intensity images presented in panel (a) pseudo-coloured according to the FRET palette defined in panel (c) reveal no detectable quenching of the donor fluorescence lifetime due to 2 h of 4OHT treatment after fixation and washing (Scale bar 10 µm). **e.** Quantification of the fraction of pixels counted as being in a FRET state (red cursor) across multiple H2B-eGFP FLIM maps acquired in DIvA nuclei in the absence of H2B-mCh confirm 4OHT treatment does not significantly change the fluorescence lifetime of H2B-eGFP after fixation or introduce artefact to detection of histone FRET (N = 6 cells, one biological replicate). **f.** Intensity images of DIvA nuclei in a single field of view co expressing H2B-eGFP and H2B-mCherry in the absence (top row) versus presence (bottom row) of 2 h of 4OHT treatment (Scale bar 20 µm). **g.** Quantification of the H2B-eGFP intensity versus H2B-mCh intensity expressed in DIvA nuclei across multiple fields of view before versus after 2 h of 4OHT treatment (left) and the resulting acceptor-donor ratio (right) (N = 22 cells, one biological replicate). **h.** Intensity images of DIvA nuclei stained with Hoechst 33342 in the absence (top) versus presence (bottom) of 2 h of 4OHT treatment (Scale bar 50 µm). **i.** Quantification of DIvA nuclear size before versus after 2 h of treatment with 4OHT (N > 100 cells, one biological replicate). Box and whisker plot shows the minimum, maximum and sample median. ns $P > 0.05$ (unpaired *t*-test).
